# CaAm-P19: a novel amelogenin-derived peptide for enamel remineralization

**DOI:** 10.1093/rb/rbag123

**Published:** 2026-06-10

**Authors:** Xiaoqian Ding, Xin Li, Cynthia Kar Yung Yiu, Zhiyi Shan

**Affiliations:** Paediatric Dentistry and Orthodontics, Faculty of Dentistry, The University of Hong Kong, Prince Philip Dental Hospital, Hong Kong 999077, China; Outpatient Department at Longfor Guangnian, The Affiliated Stomatological Hospital of Chongqing Medical University, Chongqing 401147, China; Restorative Dental Sciences, Faculty of Dentistry, The University of Hong Kong, Prince Philip Dental Hospital, Hong Kong 999077, China; Paediatric Dentistry and Orthodontics, Faculty of Dentistry, The University of Hong Kong, Prince Philip Dental Hospital, Hong Kong 999077, China; Paediatric Dentistry and Orthodontics, Faculty of Dentistry, The University of Hong Kong, Prince Philip Dental Hospital, Hong Kong 999077, China

**Keywords:** enamel remineralization, dental caries, amelogenin, biomimetic peptide, hydroxyapatite affinity

## Abstract

Dental caries remains a prevalent global oral disease characterized by the progressive demineralization of dental hard tissues. Effective remineralization strategies are necessary to arrest its progression and repair the resulting structural damage. This study investigated a novel amelogenin-derived peptide, CaAm-P19, for biomimetic enamel remineralization. *In vitro* assays evaluated the peptide’s cytocompatibility, structural stability and hydroxyapatite-binding affinity. The spatial distribution of fluorescein isothiocyanate-labeled CaAm-P19 was mapped using confocal microscopy, while overall remineralization efficacy was assessed in a demineralized bovine enamel model. Results indicated that CaAm-P19 was highly cytocompatible. BestSel deconvolution of circular dichroism spectra revealed that CaAm-P19 adopts a conformation dominated by random coils and β-sheets, and no statistically significant changes were detected following Ca^2+^ addition. Confocal imaging demonstrated continuous surface binding coupled with deep intralesional penetration exceeding 400 μm. Furthermore, the peptide attenuated calcium dissolution during demineralization challenges and directed the deposition of ordered hydroxyapatite-like crystals. Notably, CaAm-P19 achieved microhardness comparable to that of fluoride and significantly higher mineral density recovery. It successfully reconstructed a homogeneous micro-architecture, yielding a stoichiometric calcium-to-phosphorus ratio approaching that of native enamel. In conclusion, by combining deep lesion infiltration with targeted mineral nucleation, CaAm-P19 represents a scientifically robust and promising bioactive agent for early caries management.

## Graphical abstract

**Graphical Abstract:**
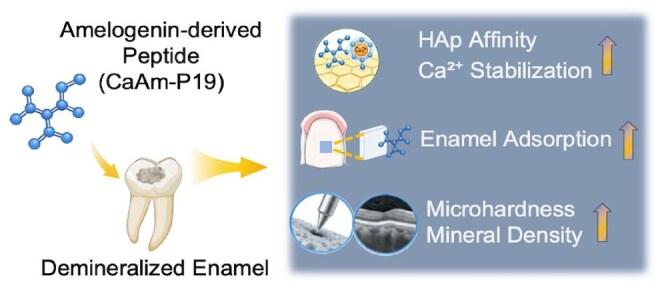

## Introduction

Dental enamel is a mineralized, non-regenerative tissue characterized by a complex prismatic structure composed of highly ordered hydroxyapatite (HAp) crystals [1]. This unique hierarchical architecture endows enamel with remarkable resistance to masticatory wear and plays a vital role in protecting the underlying dental pulp [2]. However, acidic byproducts generated by cariogenic bacterial metabolism can readily dissolve HAp crystals, leading to the formation of microscopic pores [3]. Without timely intervention, these micropores may progress into white spot lesions (WSLs), representing the earliest clinical manifestation of caries and eventually evolve into cavitated lesions that severely compromise oral health [4]. Epidemiological evidence confirms that WSLs are globally prevalent, with orthodontic patients facing a notably increased risk due to the specific challenges of maintaining adequate oral hygiene during treatment [5]. These clinical and epidemiological findings highlight an urgent need to develop enamel remineralization technologies capable of achieving deep repair and reconstructing the native ordered structure.

To counteract demineralization, various remineralization agents have been developed. Fluoride remains the most widely utilized agent, promoting remineralization by facilitating the redeposition of HAp and forming more acid-resistant fluorapatite [6]. However, fluoride lacks the biomimetic capacity to regulate oriented crystal growth and carries a risk of dental or skeletal fluorosis upon excessive exposure [7]. While HAp nanoparticles (nano-HAp) can replenish lost minerals, they exhibit a high tendency to agglomerate; this poor dispersion impedes their infiltration into demineralized micropores and prevents them from guiding the ordered arrangement of newly formed crystals [8]. Similarly, bioactive glass (BG) encounters challenges due to a mismatch between its degradation rate and mineralization kinetics. Its rapid degradation often leads to localized ion oversaturation, resulting in the precipitation of amorphous calcium phosphate rather than the functionally ordered reconstruction of the enamel lattice [9]. These limitations highlight fundamental barriers in current remineralization technologies: the inability to achieve guided, deep intralesional penetration and to orchestrate the hierarchical assembly of minerals essential for regenerating enamel function [10].

To address these limitations, biomimetic remineralization has emerged as a promising paradigm in the field of enamel repair [11]. This approach aims to simulate natural amelogenesis by employing bioactive molecules to replicate the regulation–deposition synergy inherent in the natural mineralization, thereby facilitating the precise reconstruction of enamel structure and function [12]. During the enamel formation, amelogenin, the predominant enamel matrix protein, binds specifically to the surface of HAp crystals, regulating their growth and guiding ordered arrangement along the *c*-axis, which ultimately dictates the unique prismatic structure [13]. However, utilizing natural amelogenin presents notable translational challenges. It is primarily extracted from animal tooth germs, a scarce biomaterial that demands elaborate isolation procedures, making large-scale clinical application cost-prohibitive [14]. Furthermore, its relatively large molecular weight (∼25 kDa) impedes its penetration into the narrow micropores (<100 nm in diameter) characteristic of early enamel demineralization, fundamentally limiting its efficacy in deep intralesional zones [15].

Over recent decades, research interest has gradually centered on amelogenin-derived peptides—short sequences that retain the core mineralization-active domains of natural amelogenin [16]. Engineered to facilitate scalable artificial synthesis, these fragments leverage their reduced molecular size to achieve permeability and stability, presenting significant potential for biomimetic remineralization [17, 18]. The leucine-rich amelogenin peptide (LRAP), a well-studied 59-amino-acid truncated variant, retains foundational remineralization activity by promoting HAp crystal nucleation [19]. However, its relatively long sequence still hinders deep penetration into demineralized enamel lesions, precluding comprehensive repair [20]. Another widely reported peptide, P11-4, is recognized for its ability to self-assemble into nanofibrous scaffolds that template mineral deposition [21, 22]. Yet, it lacks specific binding domains targeting demineralized enamel defects, often resulting in nonselective mineralization [23]. Therefore, a critical research gap persists in developing a biomimetic molecular tool capable of recapitulating the ‘regulated deposition’ cascade inherent to natural enamel mineralization, which is crucial for deep penetration, ordered crystal growth and functional restoration of damaged enamel.

To address this gap, we designed and synthesized a novel functional amelogenin-derived peptide, CaAm-P19 (sequence: Glu-Asp-Ser-Pro-Ala-Leu-Ile-Asp-Glu-Asp-Gly-Asp-Thr-Lys-Arg-Glu-Glu-Val-Asp). Comprising 19 amino acids with a low calculated molecular mass of ∼2.2 kDa, this peptide is engineered to readily penetrate the demineralized micropores of enamel, overcoming a major limitation of natural amelogenin, which fails to penetrate deep lesions [24, 25]. The biomimetic design of CaAm-P19 replicates the core functional domains of natural amelogenin to orchestrate targeted biomineralization. Specifically, it features a hydrophilic C-terminal motif enriched with acidic residues (glutamate and aspartate). Through carboxylate-mediated electrostatic interactions, this acidic motif promotes high-affinity binding to free calcium ions (Ca^2+^) and HAp [20, 26]. By anchoring the peptide at lattice defects, this structural feature establishes active nucleation sites, mirroring how natural enamel proteins target HAp. Furthermore, to provide the structural stability and conformational flexibility required for deep matrix infiltration, CaAm-P19 incorporates a continuous hydrophobic segment containing proline, alanine, leucine and isoleucine [27–29]. Additionally, small neutral residues (serine, glycine and threonine) are interspersed to minimize steric hindrance and maintain molecular adaptability. Through this rational sequence optimization and biomimetic structural arrangement, CaAm-P19 is engineered to achieve both deep enamel infiltration and targeted remineralization.

To evaluate this potential, the present study aims to explore the enamel adsorption and Ca^2+^-binding properties of CaAm-P19, alongside its capabilities to deeply infiltrate and restore the hardness and mineral density of subsurface enamel lesions. The null hypothesis is that CaAm-P19 has no significant influence on subsurface remineralization and exerts no effect on deep infiltration into early caries lesions *in vitro*.

## Materials and methods

Healthy bovine incisors with intact crowns and free of caries or cracks were obtained from a local compliant abattoir (Guangdong, China), cleaned of residual soft tissues and stored in physiological saline containing 0.1% sodium azide at 4°C until use. Periodontal ligament stem cells (PDLSCs) were isolated as primary cells from orthodontically extracted human premolars. Ethical approval was obtained from the Institutional Review Board (IRB) of the University of Hong Kong (Reference No. UW 24-351). The functional peptide CaAm-P19 (sequence: Glu-Asp-Ser-Pro-Ala-Leu-Ile-Asp-Glu-Asp-Gly-Asp-Thr-Lys-Arg-Glu-Glu-Val-Asp) and its fluorescein isothiocyanate (FITC)-labeled variant (FITC-CaAm-P19) were synthesized via solid-phase synthesis by TGpeptide Biotechnology Co., Ltd. (Nanjing, China). High-performance liquid chromatography (HPLC) confirmed a purity of >95% for both peptides. Liquid chromatography–mass spectrometry (LC–MS) verified their respective molecular masses (2133.14 Da for the unlabeled peptide and 2635.68 Da for the FITC-labeled peptide), which were consistent with their theoretical values, confirming structural integrity. The corresponding HPLC chromatograms and mass spectra are provided in the Supplementary Data. Other reagents utilized in this study included HAp (≥99% purity, 50 nm, Sigma-Aldrich), a Cell Counting Kit-8 (CCK-8; Yeason, Japan), analytical-grade calcium chloride (CaCl_2_), disodium hydrogen phosphate (Na_2_HPO_4_) and acetic acid (CH_3_COOH; Sinopharm, China), as well as electron microscopy grade 25% glutaraldehyde fixative (Solarbio, Beijing, China).

### Cell viability assay

The CCK-8 assay was employed to evaluate the cytocompatibility of CaAm-P19. Human PDLSCs (hPDLSCs) were selected for preliminary assessment as these oral-derived mesenchymal stem cells are highly sensitive to biomaterial cytotoxicity, possess well-established culture protocols and are widely utilized for the initial safety screening of dental remineralization materials [30]. Cells at passages 3–5 were cultured in Dulbecco’s Modified Eagle Medium (DMEM) supplemented with 10% fetal bovine serum (FBS) and 1% penicillin–streptomycin and maintained at 37°C in a humidified 5% CO_2_ atmosphere. Following enzymatic detachment, the cell suspension was adjusted to a density of 5 × 10³ cells per well. Approximately 100 μL of this suspension was seeded into each well of a 96-well plate and incubated for 24 h to allow for cell adhesion. The original medium was then aspirated and replaced with 100 μL of fresh medium containing CaAm-P19 at varying concentrations (6.25, 12.5, 25, 50 and 100 μM). This concentration gradient was selected in accordance with previously published studies evaluating analogous peptides for dental hard tissue remineralization [31]. A blank control group (medium only, no cells) and an untreated control group (hPDLSCs in standard medium) were included. All experimental conditions were tested using three independent biological replicates, with three technical replicate wells per group. After incubation periods of 1, 6, 12, 24 and 48 h, 10 μL of CCK-8 reagent was added to each well, followed by an additional 2 h of incubation. Finally, the absorbance at 450 nm (OD450) was measured using a microplate reader.

### CD spectroscopy

To analyze the secondary structure characteristics of the peptide CaAm-P19 and the effect of Ca^2+^ on its conformation, circular dichroism (CD) spectroscopy was employed, using a J-815 spectropolarimeter (JASCO Corporation, Tokyo, Japan). Following a 30-min instrument warm-up and baseline calibration, a blank 0.1 mM HEPES buffer was scanned under identical conditions for background subtraction. All measurements were conducted at 25°C using a quartz cuvette with a 1-mm optical path length. The CaAm-P19 peptide was first dissolved in 0.1 mM HEPES buffer and magnetically stirred for 30 min. Subsequently, the solution was passed through a 0.22 µm organic-phase filter to eliminate undissolved particulates and the filtrate was diluted to a final concentration of 0.2 mg/mL. To assess how Ca^2+^ modulates the peptide’s conformation, the CaAm-P19 solution was combined with a 3.3 mM CaCl_2_ solution in a 1:1 ratio. The resulting mixture was incubated in a shaking incubator at 37°C for 30 min to allow for sufficient interaction. For spectral acquisition, the measurement range was set from 190 to 300 nm, with a scan speed of 100 nm [32].

### HAp binding assay

To evaluate the binding affinity of the peptide to HAp, CaAm-P19 at varying concentrations (6.25, 12.5, 25, 50 and 100 μM) was mixed with 0.4 mg of HAp nanocrystals (specific surface area: 30 m^2^/g) and incubated at 37°C for 2 h. All experimental conditions were tested using three independent replicates with three technical replicate wells per group. To quantify peptide binding, the concentration of free CaAm-P19 before and after HAp exposure was determined from the sample supernatant using a Micro Bicinchoninic Acid (BCA) Protein Assay Kit (Solarbio, Beijing, China). The adsorption data were modeled using the Langmuir adsorption isotherm, expressed in its linear form as: 1/*Q *= 1/*N *+ 1/(*K *×* N* × *C*_eq_), where *Q* represents the amount of peptide bound per square meter of HAp surface (mol/m^2^), *N* is the maximum adsorption capacity of HAp per square meter (mol/m^2^), *C*_eq_ denotes the equilibrium concentration of free peptide (mol/L) and *K* stands for the affinity constant of CaAm-P19 for the HAp surface (L/mol). The affinity constant (*K*) and maximum adsorption capacity (*N*) were calculated from the slope and intercept, respectively, of the linear regression plot of 1/*C*_eq_ versus 1/*Q*.

### Ca^2+^ release inhibition rate

To simulate a cariogenic acidic environment, CaAm-P19 solutions (6.25, 12.5, 25, 50 and 100 μM) were prepared in an acetic acid solution (pH 4.5). For the assay, 0.4 mg of HAp powder was added to 2 mL of each peptide solution. The suspensions were vortexed to ensure uniform mixing and incubated in a constant-temperature water bath at 37°C for 2 h. A blank control group, consisting of 0.4 mg of HAp in 2 mL of peptide-free acetic acid solution (pH 4.5), was processed in parallel. All experimental conditions and the blank were tested using three independent replicates with three technical replicates per group. Following incubation, the mixtures were centrifuged at 10 000 rpm and 4°C for 10 min. A 1-mL aliquot of the supernatant was collected from each sample, and the pH was adjusted to 7.4 to meet the operational requirements of the detection kit. The concentration of free calcium ions in the neutralized supernatants was then measured using a Calcium Ion Assay Kit (Beyotime, Shanghai, China). The calcium ion concentration of the blank group served as the baseline release value (*C*_0_). The inhibition rate was calculated using the following formula:


$$\mathrm{Inhibition}\;\mathrm{rate}\;(\%)=(1-C_{\text{exp}}/C_{0})\times 100\%,$$

where *C_exp_* is the Ca^2+^ concentration of the peptide-treated experimental group.

### TEM, SAED and HRTEM analysis of calcium phosphate mineralization

Transmission electron microscopy (TEM) combined with selected area electron diffraction (SAED) and high-resolution TEM (HRTEM) was used to investigate the binding of the peptide to calcium phosphate particles. To simulate HAp precursors, a solution with a Ca/P molar ratio of ∼1.67 was prepared in a sterile microcentrifuge tube using ultrapure water, with CaCl_2_ and Na_2_HPO_4_ adjusted to final concentrations of 3.3 and 1.6 mM, respectively. This solution was divided into two groups: a blank control containing only the calcium and phosphate salts, and a peptide treatment group supplemented with 25 μM CaAm-P19. All experiments were conducted using three independent replicates, with three parallel samples per condition at each time point. After incubation at 37°C for either 30 min or 24 h, 10-μL aliquots of each sample were drop-cast onto 300-mesh carbon-coated copper grids and air-dried at room temperature. Morphological imaging was performed using an FEI Talos F200X TEM (Hillsboro, USA) operated at an accelerating voltage of 200 kV and a working distance of 10 mm. SAED analysis was subsequently conducted on the same instrument to collect diffraction patterns from the calcium phosphate particles. The resulting diffraction rings were indexed to identify specific crystal planes (e.g., (0 0 2), (2 1 1) and (0 0 4)) for structural characterization.

### Demineralization and remineralization

**Figure 1:**
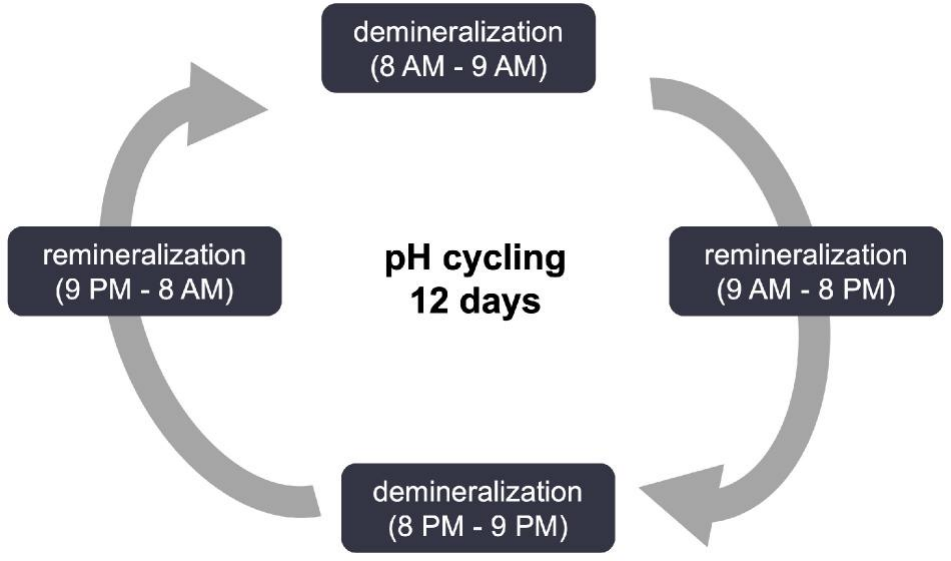
Schematic representation of the experimental design and dynamic pH cycling protocol.

Enamel samples were assigned to one of five experimental groups: Control (healthy enamel), Demin (demineralization only, no remineralization), Remin (standard remineralization without active agents), Fluoride (remineralization supplemented with NaF) and Peptide (remineralization supplemented with CaAm-P19). To induce artificial caries lesions, all groups except the healthy control were immersed in a demineralization solution (50 mM acetic acid, 2.2 mM Ca(NO_3_)_2_, 2.2 mM KH_2_PO_4_, 5.0 mM NaN_3_ and 0.5 ppm NaF; pH 4.5) for 3 days, with the solution replaced daily. Following initial demineralization, the three remineralization groups underwent a pH cycling protocol for 12 days (Figure 1). Each 12-h cycle consisted of 1 h of demineralization followed by 11 h of remineralization. The model is designed to simulate dynamic demineralization and remineralization cycles within the oral cavity to evaluate the biomimetic repair efficacy of the treatments. A basal remineralization solution was prepared according to a standard formula (20 mM HEPES, 0.9 mM KH_2_PO_4_, 1.5 mM CaCl_2_, 130 mM KCl and 1.0 mM NaN_3_; pH 7.4). For the Remin group, this basal solution was used as-is. For the treatment groups, the basal solution was supplemented with either 1.0 mM NaF (Fluoride group) or 25 μM CaAm-P19 (Peptide group), with all other components and pH kept constant [33].

### CLSM analysis of CaAm-P19 adsorption and infiltration

Healthy enamel samples were prepared by cutting bovine enamel into 3 × 3 × 1 mm blocks. These were polished sequentially with 400–2000-grit silicon carbide (SiC) paper under running water for 30 s per grade [34]. Two sets of experiments were performed to separately evaluate the surface adsorption and deep infiltration of CaAm-P19 within the enamel. For the surface adsorption assay, acid-etched demineralized enamel was prepared by applying 37% phosphoric acid evenly onto the enamel surface for 30 s. Both healthy and acid-etched enamel samples were immersed in a 25 μM FITC-labeled CaAm-P19 solution and incubated at 37°C in the dark for 30 min. To remove unadsorbed peptides, the samples were gently rinsed three times with PBS (1 min per wash). For the deep infiltration assay, five surfaces of the healthy enamel blocks were sealed with acid-resistant nail varnish, leaving only the top enamel surface exposed. These samples were immersed in a demineralization solution for 3 days to establish an enamel demineralization model. The specimens were then incubated in the 25 μM FITC-CaAm-P19 solution and rinsed following the exact same protocol described above. Afterward, the blocks were longitudinally sectioned at the midpoint perpendicular to the exposed enamel surface to allow for cross-sectional observation. Imaging was performed using a Zeiss LSM 880 confocal laser scanning microscope (CLSM; Carl Zeiss, Germany) equipped with a 40 × water-immersion objective (numerical aperture = 1.2). Fluorescence was captured using an excitation wavelength of 488 nm and an emission wavelength of 525 nm. For the surface assay, images were acquired at the top enamel surface as well as at a depth of 20 μm below the surface. For the deep infiltration assay, the longitudinal sections were imaged to assess the in-depth spatial distribution of CaAm-P19 inside the demineralized lesions. Both assays were conducted across three independent experiments, with three parallel enamel specimens prepared for each group (healthy, acid-etched and demineralized) per run.

### XRD analysis of enamel remineralization

To analyze the crystal structure of enamel samples following different treatments, X-ray diffraction (XRD) was employed. The five previously described experimental groups (three replicates per group) were subjected to 12 days of pH cycling. Afterward, the enamel specimens from each group were ground into a fine powder with a particle size of ≤5 μm. XRD analysis was performed using a D8 Venture (Bruker, Germany) equipped with Cu Kα radiation (λ = 0.154 nm). The instrument was operated at a tube voltage of 40 kV and a tube current of 40 mA. Scans were conducted over a 2*θ* range of 20°–40° to capture the characteristic peak interval of HAp. The scanning parameters included a step size of 0.02° and a scanning speed of 2°/min. The resulting XRD patterns were processed using Bruker EVA software. Specifically, the position, intensity and full width at half maximum (FWHM) of the primary HAp characteristic peaks (2*θ* = 25.9°, 31.8° and 32.9°) were recorded for structural analysis.

### SMH evaluation

Six samples were assigned to each experimental group and sectioned into blocks measuring 3 × 3 × 2 mm. For each sample, surface microhardness (*SMH*) was evaluated at three distinct time points: baseline hardness of the healthy enamel (*SMH_0_*), hardness after 3 days of initial demineralization (*SMH*_1_) and final hardness after 12 days of pH cycling for remineralization (*SMH*_2_). Measurements were performed using an INNOVATEST FALCON 507 Vickers microhardness tester (Maastricht, the Netherlands) applying a loading force of 200 gf (1.96 N) with a dwell time of 10 s. At each time point, 10 measurement indentations were evenly distributed across the sample surface. To avoid edge effects and overlapping stress fields, a minimum distance of 200 μm was maintained between adjacent points and all visible defects were avoided. The Vickers hardness value (HV) of each point was recorded and the average value per sample was calculated. To compare the remineralization efficacy among the different groups, the percentage of surface microhardness recovery (*SMHR*) was computed using the following formula:


$$\mathrm{SMHR}\;(\%)=[({\mathrm{SMH}}_{2}-{\mathrm{SMH}}_{1})/({\mathrm{SMH}}_{0}-{\mathrm{SMH}}_{1})]\times 100\%.$$

### Analysis of enamel 3D mineralization parameters by micro-CT

Three specimens were prepared for each experimental group and sectioned into rectangular blocks measuring 6 × 2 × 2 mm. To isolate the solutions, five surfaces of each block were coated with acid-resistant nail varnish, leaving only the top enamel surface exposed. This exposed surface was equally divided along its length into three 1 × 1 mm regions: Control (left), Demin (middle) and Remin (right). First, the Control region was sealed with nail varnish, while the Demin and Remin regions were left exposed and immersed in a demineralization solution for 3 days to establish the demineralized lesions. The samples were then rinsed with ultrapure water and air-dried. Next, the Demin region was sealed with varnish, leaving only the Remin region exposed. The samples were subsequently immersed in their respective treatment solutions for 12 days of pH cycling. Micro-computed tomography (Micro-CT) analysis was performed using a SkyScan 1267 system (Bruker, Kontich, Belgium). The scanning parameters were set to a voltage of 50 kV, a current of 200 μA, an isotropic pixel size of 5 μm and an exposure time of 800 ms. Scans were performed over the entire sample volume. Three-dimensional images were reconstructed using NRecon software (Bruker), and each specific region was delineated as a region of interest (ROI) using CTAn software (Bruker). Prior to enamel mineral density quantification, HAp calibration was performed to establish a standard curve, ensuring the accuracy of subsequent density measurements. The enamel mineral density percent recovery (*EMDR*) was calculated to evaluate the efficiency of remineralization. *EMDR* was computed using the following formula:


EMDR (%)=[(MD_remin-MD_demin)/(MD_health-MD_demin)]×100%,

where *MD_health* is the mineral density of healthy enamel (baseline), *MD_demin* is the mineral density after demineralization and *MD_remin* is the mineral density after remineralization. This formula represents the percentage of mineral loss that was recovered during the remineralization phase.

### SEM with EDS for morphological and elemental analysis

Three independent enamel samples per experimental group were prepared for morphological observation via scanning electron microscopy (SEM) and elemental analysis via energy-dispersive spectroscopy (EDS). The samples were sectioned into 3 × 3 × 2 mm blocks and subjected to the demineralization and remineralization treatments. To remove surface impurities, the blocks were first ultrasonically cleaned in ultrapure water (300 W, 5 min). Subsequently, the samples were fixed in a 2.5% glutaraldehyde solution at 4°C for 24 h to preserve surface morphology. Dehydration was carried out through a graded ethanol series (30%, 50%, 70%, 80%, 90%, 95% and 100%) for 15 min per step. After drying in a critical point dryer (K850, Quorum Technologies, East Sussex, UK), a 5–10 nm thick gold film was applied to the sample surfaces using an ion sputter coater (E-1045, Hitachi, Tokyo, Japan). Imaging was performed using an S3400N VP SEM (Hitachi, Tokyo, Japan) operating at an accelerating voltage of 10 kV and a working distance of 8 mm. Surface morphology images were captured at a magnification of 1000×. For EDS point scanning, three spatially distinct regions were randomly selected on each sample to determine the atomic percentages (At%) of calcium (Ca), phosphorus (P), carbon (C), oxygen (O) and fluorine (F).

### Statistical data analysis

Data are presented as the mean ± standard deviation (SD). Statistical analyses were performed using SPSS software (version 26.0; IBM Corp., Armonk, NY, USA). A one-way analysis of variance (ANOVA) was applied to assess differences among multiple experimental groups, followed by the Bonferroni *post hoc* test for pairwise comparisons. The level of statistical significance was set at *P *< 0.05. All graphs were generated using Origin 2025b SR1 software (OriginLab, Northampton, MA, USA).

## Results

### Cell viability of CaAm-P19

**Figure 2:**
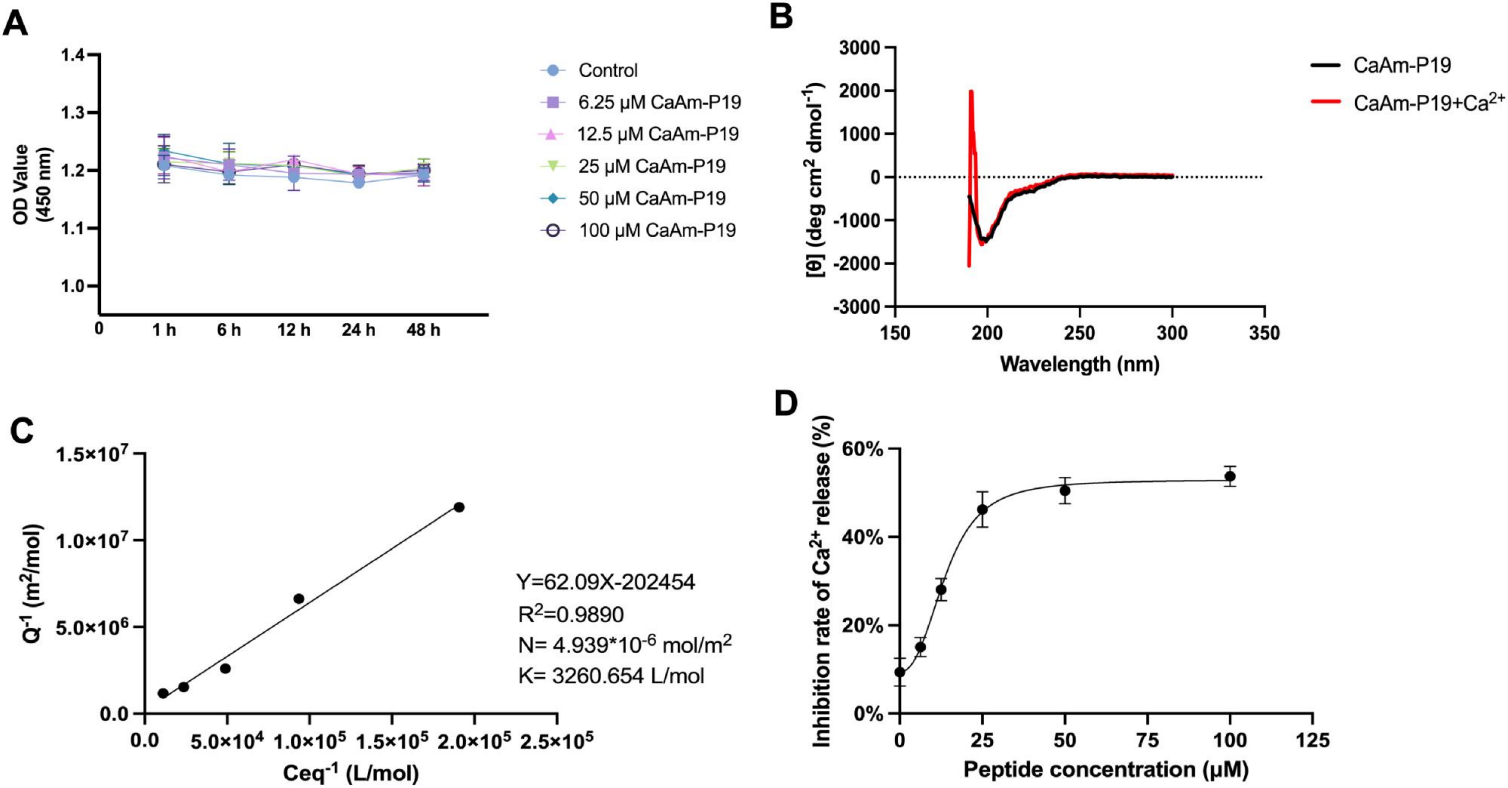
Biocompatibility, structural stability and functional characterization of CaAm-P19. (**A**) Viability of periodontal ligament stem cells treated with CaAm-P19 (6.25–100 μM) for 1-48 h (CCK-8 assay). No significant cytotoxicity was observed (*P *> 0.05 vs. control). (**B**) CD spectra of CaAm-P19 in 0.1 mM HEPES buffer and with 3.3 mM Ca^2+^. No significant conformational shifts were detected (*P *> 0.05). (**C**) Linearized Langmuir adsorption isotherm of CaAm-P19 binding to HAp (1/*Q* vs 1/*C_eq_*). The excellent fit (*R*^2^ = 0.9890) yielded an affinity constant *K *= 3.26 × 10³ L/mol and maximum adsorption capacity *N *= 4.94 × 10^−6 ^mol/m^2^. (**D**) Dose-dependent inhibition of Ca^2+^ release from HAp at pH 4.5, showing a marked increase up to 25 μM and approaching saturation at higher concentrations. (*For all panels, n = 3 independent biological replicates with three technical replicates or scans per group*).

As shown in Figure 2A, PDLSCs exposed to CaAm-P19 at 6.25, 12.5, 25, 50 and 100 µM for 1, 6, 12, 24 and 48 h exhibited OD values comparable to the control group. This suggests that there were no significant decreases or fluctuations in cell viability across all tested concentrations and time points (*P *> 0.05).

### CD spectroscopy

CD spectroscopy was employed to characterize the secondary structure of CaAm-P19 in the absence and presence of calcium ions, with spectra recorded over a wavelength range of 190–300 nm (Figure 2B). The native CaAm-P19 in 0.1 mM HEPES buffer and the calcium-incubated peptide both exhibited a distinct negative ellipticity minimum at ∼198 nm, followed by a transition to positive ellipticity. Notably, no significant shifts in peak position or intensity were observed between the two conditions. Because calcium was supplemented as CaCl_2_, the strong UV absorbance of chloride ions below 195 nm resulted in an anomalous positive fluctuation in the red curve. This represents an instrumental artifact rather than a genuine structural alteration of the peptide. Although the raw spectra are presented in their entirety to ensure data transparency, subsequent structural analyses were restricted to valid signals above 195 nm. Quantitative deconvolution of the spectra using BestSel software (Supplementary Table S1) revealed that in the calcium-free state, the secondary structure consisted of *β*-sheets (30.4 ± 2.3%), *β*-turns (14.6 ± 2.3%), *α*-helices (8.2 ± 1.3%) and random coils (46.8 ± 2.6%). Following calcium addition, these proportions remained consistently stable: *β*-sheets (32.1 ± 4.4%), *β*-turns (14.5 ± 0.9%), *α*-helices (7.9 ± 1.6%) and random coils (45.5 ± 3.4%). No statistically significant differences were detected for any secondary structural component before and after calcium addition (*P *> 0.05), indicating that the overarching secondary conformation of CaAm-P19 is stably maintained in the presence of calcium ions under the tested conditions.

### HAp-binding ability of CaAm-P19

The binding of CaAm-P19 to HAp was analyzed using the Langmuir adsorption isotherm. The linearized plot of 1/*Q* versus 1/*C*_eq_ demonstrated an excellent fit (*R*^2^ = 0.9890), where *Q* represents the amount of bound peptide per unit surface area of HAp, and *C_eq_* is the equilibrium concentration of the free peptide (Figure 2C). Calculations derived from this model yielded an affinity constant (*K*) of 3260.65 L/mol, indicating a robust binding interaction. Furthermore, the maximum adsorption capacity (*N*) was determined to be 4.94 × 10^−6 ^mol/m^2^, reflecting a substantial accumulation of the peptide on the HAp surface.

### Inhibition of Ca^2+^ release

The capacity of CaAm-P19 to inhibit Ca^2+^ release from HAp in an acidic environment (pH 4.5) was evaluated across varying peptide concentrations (Figure 2D). The inhibition rate exhibited a progressive, concentration-dependent increase. Specifically, the inhibitory effect rose sharply up to 25 μM, whereas further enhancement became marginal at higher concentrations (≥50 μM). This trend demonstrates a robust dose-dependent protective effect that approaches saturation at higher doses.

### CLSM analysis of CaAm-P19 adsorption and infiltration

**Figure 3:**
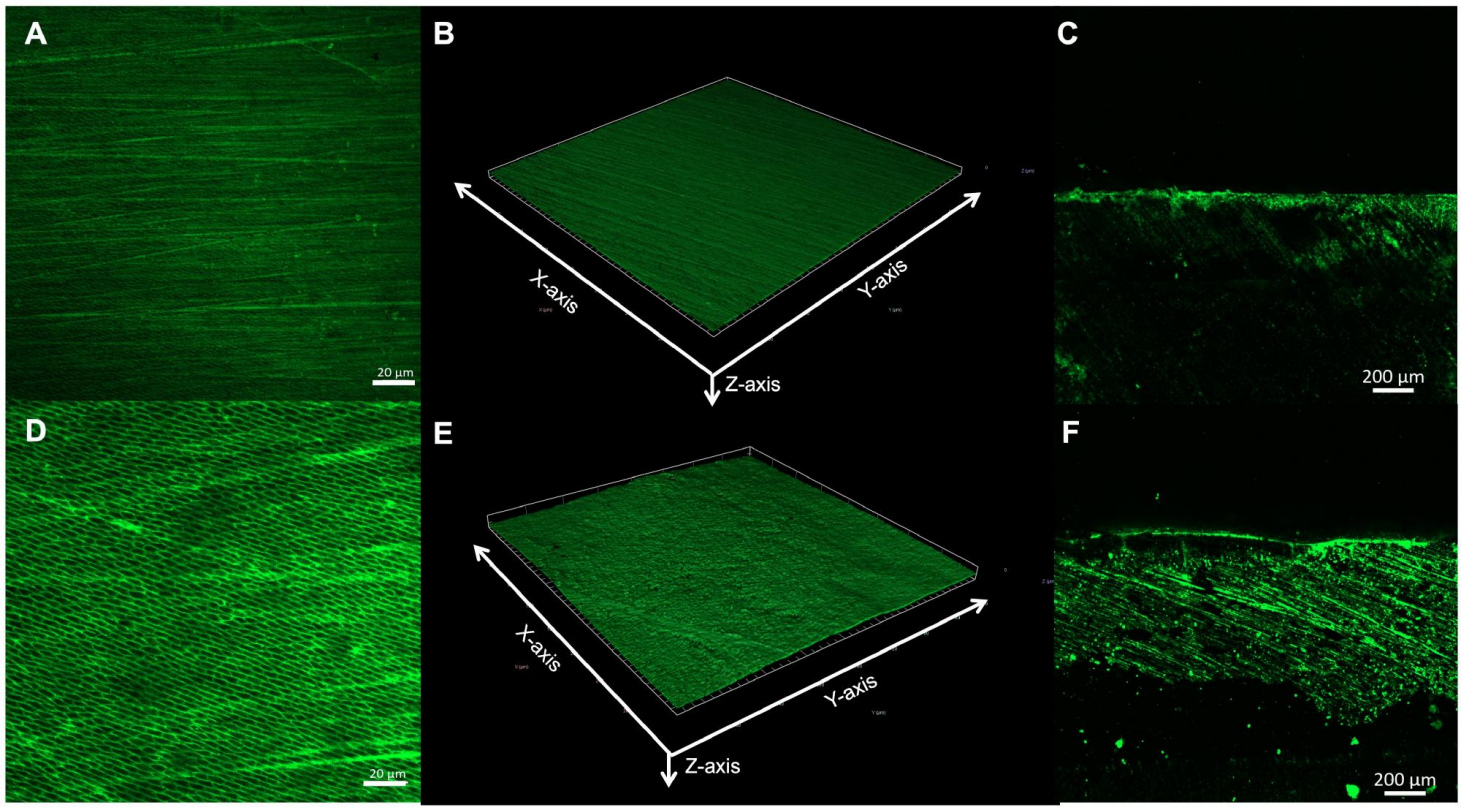
CLSM imaging of FITC-labeled CaAm-P19 adsorption and infiltration in bovine enamel. Representative 2D and 3D images of peptide surface adsorption on (**A** and **B**) healthy enamel and (**D** and **E**) acid-etched enamel (37% phosphoric acid, 30 s). Longitudinal sections showing the infiltration depth of the peptide in (**C**) healthy enamel and (**F**) 3-day demineralized enamel. (*n *= 3 independent experiments with three parallel specimens per group).

CLSM was employed to observe the surface adsorption and internal infiltration of FITC-labeled CaAm-P19 (25 μM) in bovine enamel. Both healthy and acid-etched (37% phosphoric acid for 30 s) enamel surfaces displayed homogeneous fluorescence, indicating an even distribution of the adsorbed peptide. Representative 2D and 3D images visually confirmed this uniform surface coating on both healthy (Figure 3A and B) and etched (Figure 3D and E) enamel. To assess penetration capacity, longitudinal sections were examined. In healthy enamel, CaAm-P19 infiltrated to a depth of ∼50 μm (Figure 3C). In contrast, in enamel subjected to a 3-day demineralization protocol, the peptide penetrated significantly deeper, reaching depths exceeding 400 μm (Figure 3F).

### TEM, SAED and HRTEM analysis of calcium phosphate mineralization

**Figure 4:**
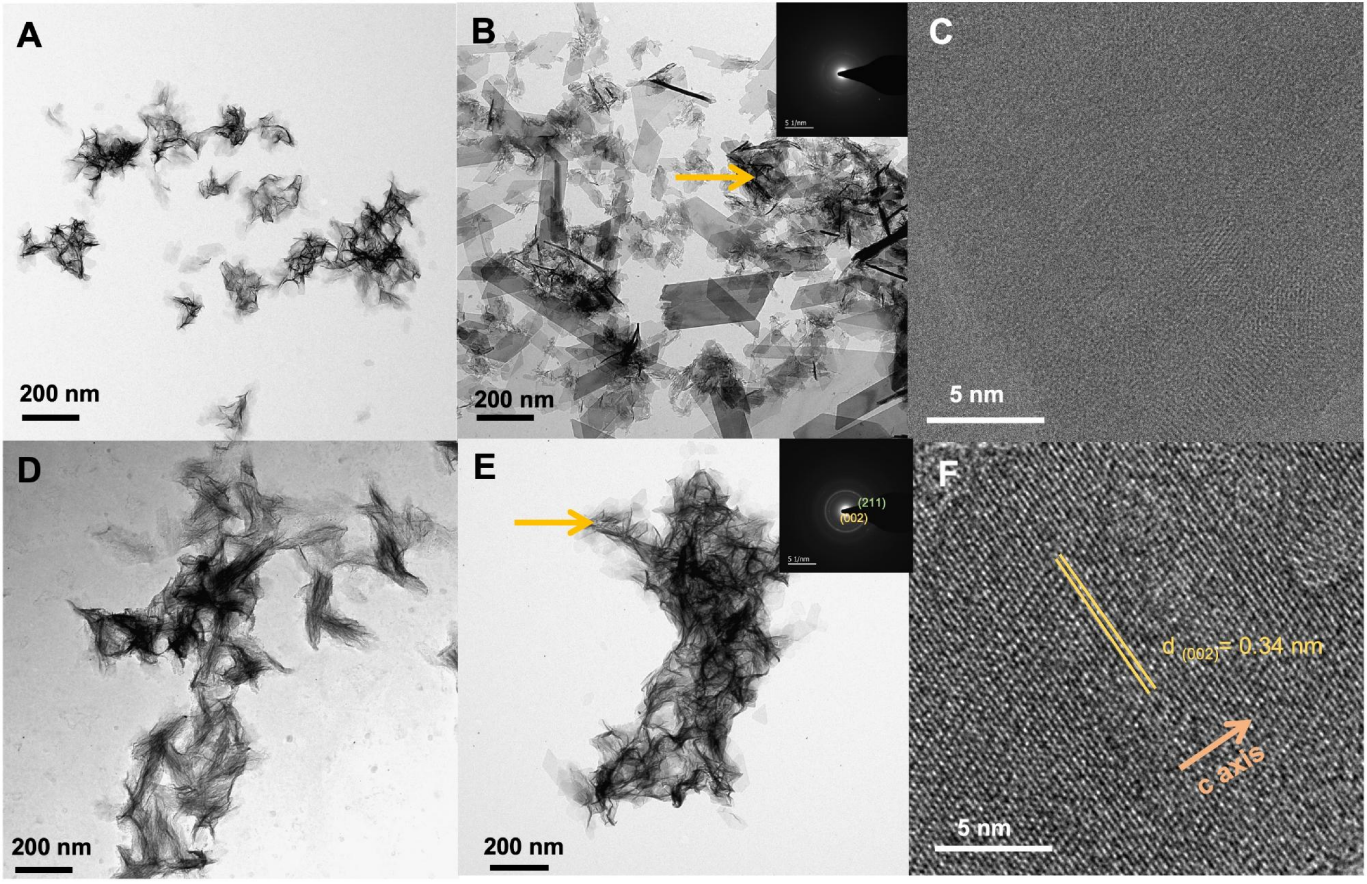
TEM, SAED and HRTEM characterizations of calcium phosphate precipitates formed in HAp precursor solutions in the absence (**A–C**) or presence (**D–F**) of CaAm-P19 (25 μM). (**A** and **B**) TEM images of the control group after 30 min and 24 h of incubation, respectively. The corresponding SAED pattern (in **B**) confirms an amorphous state with no distinct diffraction rings. (**C**) HRTEM image of the control group lacking ordered lattice fringes. (**D** and **E**) TEM images of the peptide-treated group at 30 min and 24 h, respectively, displaying organized crystalline assemblies. The SAED pattern (in **E**) exhibits clear diffraction rings indexed to the HAp (0 0 2) and (2 1 1) planes. (**F**) HRTEM image of the peptide group showing distinct crystal bundles aligned along parallel *c*-axes, with a *d*-spacing of 0.34 nm corresponding to the HAp (0 0 2) plane. (*n *= 3 independent experiments).

TEM was utilized to evaluate the influence of CaAm-P19 (25 μM) on the morphological evolution of calcium phosphate precipitates derived from HAp precursor solutions. Over incubation periods of 30 min and 24 h, stark structural differences emerged between the two groups (Figure 4). The control group yielded irregular, scattered and amorphous precipitates lacking uniform and ordered structure features at both time points (Figure 4A and B). Conversely, the presence of the peptide induced the formation of well-organized, hierarchical calcium phosphate assemblies (Figure 4D and E), demonstrating the capacity of CaAm-P19 to template and guide mineralization.

Crystallographic analysis via SAED further highlighted these differences. While the control group lacked discernible diffraction rings, confirming an amorphous state (Figure 4B, inset/SAED), the peptide-treated group displayed prominent rings indexed to the (0 0 2) and (2 1 1) planes, characteristic of crystalline HAp (Figure 4E, inset/SAED). HRTEM provided deeper ultrastructural insights. The peptide group revealed distinct crystal bundles aligned along the parallel *c*-axes, with a measured lattice spacing (*d*-spacing) of 0.34 nm corresponding to the HAp (0 0 2) plane (Figure 4C). As expected, no ordered lattice fringes were detected in the control group (Figure 4F).

### XRD analysis of enamel remineralization

**Figure 5:**
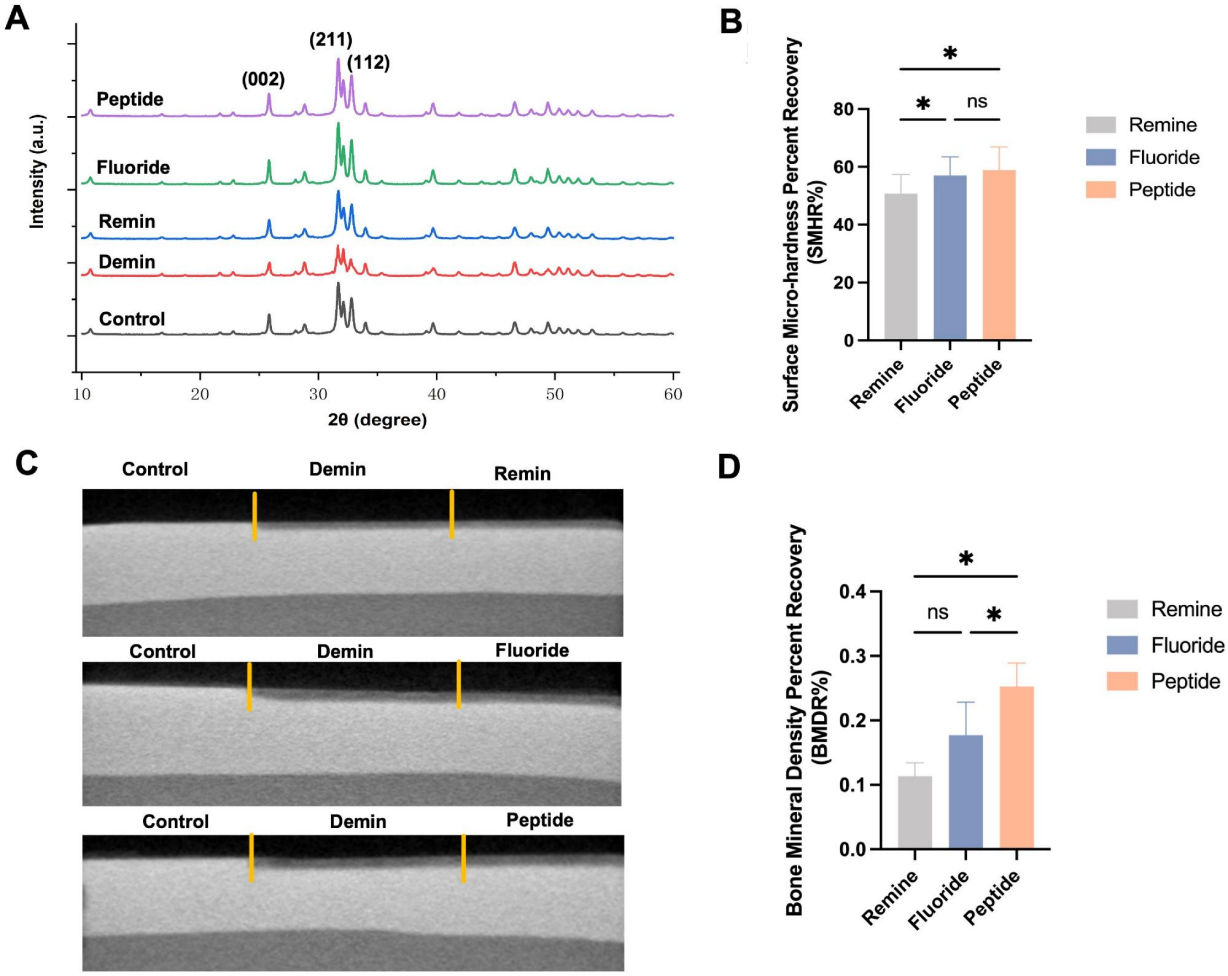
Compositional, mechanical and microstructural characterization of enamel remineralization across five experimental groups. (**A**) XRD patterns of the enamel specimens (*n *= 3). Characteristic HAp reflections are marked at 2*θ* = 25.9° (0 0 2), 31.8° (2 1 1) and 32.0° (1 1 2). Note the pronounced attenuation of peak intensities in the demineralized group and their subsequent recovery following 12 days of remineralization treatments. (**B**) SMH assessments across the three experimental stages (baseline, post-demineralization and post-remineralization) and the corresponding *SMHR*. Both the peptide and fluoride groups exhibited significantly higher SMHR than the remineralization solution alone (*P *< 0.05), with no statistical difference between the two (*P *> 0.05) (*n *= 6 specimens, 10 measurement points/specimen). (**C**) Representative Micro-CT evaluations illustrating the three-dimensional mineralization profiles of the enamel specimens (*n *= 3 independent replicates). (**D**) Quantitative analysis of *EMDR* among the treatment groups. Data were HAp-calibrated for densitometric accuracy. The peptide group demonstrated a significantly higher *EMDR* compared to the fluoride and remineralization solution groups (*P *< 0.05).

XRD was employed to evaluate the crystallographic phase and structural integrity of the enamel across five experimental groups. All acquired spectra were normalized to facilitate the direct comparison of peak intensities. Compared to healthy enamel, the demineralized group exhibited a pronounced reduction in the intensity of two primary HAp diffraction peaks at 2*θ* = 25.9° and 31.8°, corresponding to the (0 0 2) and (2 1 1) reflections, respectively (Figure 5A). This significant signal attenuation reflects the severe degradation of the HAp crystalline lattice induced by the demineralization process. However, following a 12-day pH cycling regimen, all remineralization groups demonstrated a noticeable recovery in the intensity of these characteristic HAp peaks, indicating successful crystalline repair and mineral deposition.

### SMH evaluation

The SMH of the enamel specimens was assessed across five groups at three distinct stages: baseline, post-demineralization and post-remineralization. Following the 12-day treatment regimen, the *SMHR* was calculated (Figure 5B). The peptide-treated group achieved the highest *SMHR*, followed by the fluoride-treated group; both demonstrated a significantly greater recovery than the remineralization solution alone (*P *< 0.05). Notably, while the peptide group exhibited a marginally higher mean *SMHR* than the fluoride group, the difference lacked statistical significance (*P *> 0.05), indicating a remineralization efficacy comparable to that of the fluoride standard.

### Micro-CT evaluation of three-dimensional mineralization

Micro-CT was employed to evaluate the 3D mineralization profiles of the enamel specimens, with each sample partitioned into three predefined regions of interest: Control, Demin and Remin. Throughout the experiment, the Control region maintained a stable MD values across all the groups. Conversely, the Demin region exhibited pronounced demineralization features, primarily evidenced by a marked reduction in MD. Within the Remin region, however, the extent of mineral recovery varied significantly among the treatments (Figure 5C and D). The peptide-treated group achieved the highest MD recovery rate, significantly outperforming both the remineralization solution alone and the fluoride-treated group (*P *< 0.05). Notably, no statistical difference in MD recovery was observed between the fluoride group and the remineralization solution (*P *> 0.05), further highlighting the superior remineralization efficacy of the peptide within the deep enamel lesions.

### SEM with EDS for morphological and elemental analysis

**Figure 6:**
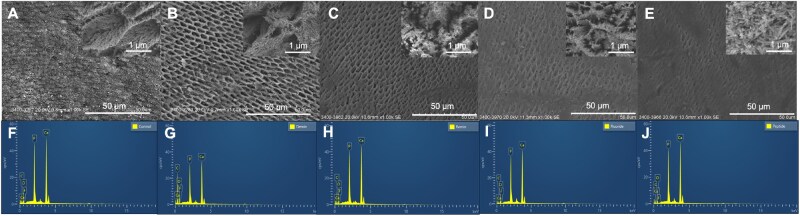
Surface micromorphology and elemental composition of enamel specimens across the experimental groups. (**A–E**) Representative SEM micrographs captured at 1000× and 10 000× magnifications. (**A**) Untreated control, demonstrating an intact, dense native architecture with occluded enamel prisms. (**B**) Demineralized group, exhibiting severe mineral loss characterized by a fragmented topography and completely exposed prism cores. (**C–E**) Enamel surfaces following treatment with (**C**) the remineralization solution alone, (**D**) fluoride and (**E**) the peptide. Note the progressive structural recovery across the treatments, with the peptide group achieving the most profound biomimetic restoration, effectively sealing the inter-prismatic spaces to closely approximate the native healthy morphology. (**F–J**) Corresponding EDS spectra mapping the elemental distributions (C, O, F, Ca and P) across the respective groups. (*n *= 3 independent specimens per group, with three randomly selected regions of interest analyzed per specimen; detailed quantitative EDS data and stoichiometric ratios are provided in Supplementary Table S2).

SEM evaluation of enamel surface morphology revealed distinct microstructural alterations across the experimental groups (Figure 6). The Control group (Figure 6A) displayed an intact, smooth topography characterized by well-organized enamel prisms and occluded inter-prismatic spaces, typical of healthy native enamel. Conversely, the Demin group (Figure 6B) exhibited pronounced surface erosion and microstructural disorganization, evidenced by the severe exposure of enamel prism cores and widened inter-prismatic gaps. Following treatment, all remineralization groups (Figure 6C–E) demonstrated varying degrees of morphological recovery, indicated by reduced prism exposure and partial restoration of surface continuity. Notably, the peptide-treated group (Figure 6E) exhibited the most profound biomimetic repair. Its surface architecture closely approximated that of the healthy control, featuring a dense, continuous mineralized overlayer and substantially smoothed topographical contours.

These morphological observations were quantitatively corroborated by EDS (Figure 6F–J). Among the treated cohorts, the peptide group (Figure 6J) yielded the highest weight percentages (wt%) of calcium (Ca) and phosphorus (P). Crucially, its Ca/P molar ratio (1.69 ± 0.16) almost identically matched the theoretical stoichiometric value of ideal HAp (1.67). Furthermore, this ratio was statistically comparable to that of the intact healthy control (1.79 ± 0.15, *P *> 0.05) (Supplementary Table S2), confirming the deposition of a compositionally mature and highly crystalline apatite phase.

## Discussion

This study set out to test the null hypothesis that CaAm-P19 would yield no significant improvement in subsurface mineral recovery and exhibit no detectable infiltration into deep demineralized lesions under *in vitro* conditions. Based on our findings, this null hypothesis is firmly rejected. Mechanically, HAp adsorption and calcium ion release inhibition assay confirmed that CaAm-P19 possesses a robust affinity for HAp surfaces, effectively reducing local calcium liberation from demineralized enamel, a behavior consistent with acidic motif-containing peptides [35]. This targeted binding was spatially corroborated by CLSM. While immediate surface adsorption was observed on acid-etched enamel, the peptide successfully penetrated to depths exceeding 400 μm in the 3-day deep demineralization model, providing direct *in vitro* evidence of profound subsurface infiltration.

A plausible structural basis for this deep infiltration was elucidated via CD spectroscopy. Quantitative secondary structure deconvolution (BestSel) revealed that random coils and *β*-sheets constitute the predominant conformational components of CaAm-P19. The high proportion of random coils (46.8 ± 2.6%) confers substantial conformational flexibility. Combined with its low molecular mass (≈2.2 kDa), this flexibility significantly reduces the hydrodynamic radius and steric hindrance of the peptide, thereby facilitating diffusion through the narrow, tortuous microporosities of demineralized enamel, a diffusion profile similarly observed for short, unstructured peptides that traverse biological barriers [24]. Concurrently, the appreciable *β*-sheet content likely imparts local structural order, ensuring conformational stability [36]. Crucially, the addition of Ca^2+^ induced no statistically significant structural shifts, indicating that CaAm-P19 maintains its global conformation in calcium-rich microenvironments. This structural conservation may allow the peptide to remain stably anchored to demineralized enamel substrates while retaining the flexibility required for biomineralization interactions. However, CD spectroscopy only reflects global secondary structure and cannot independently resolve the molecular-level interactions between CaAm-P19 and Ca^2+^. Following the peptide-guided mineralization process, EDS and XRD analyses confirmed that the regenerated neo-crystals exhibited a Ca/P molar ratio approximating the stoichiometric value of natural HAp, retaining characteristic HAp diffraction profiles [37, 38]. Furthermore, TEM observation revealed that the precipitated calcium phosphate aggregates assembled into organized architectures under the regulatory influence of CaAm-P19.

The rationally designed sequence of CaAm-P19 establishes a mechanistic framework for its biomimetic efficacy. As a truncated peptide, CaAm-P19 possesses a low molecular weight that circumvents the steric hindrance that typically restrict the diffusion of full-length enamel matrix proteins, allowing deep lesion penetration. Once localized within the subsurface micropores, the acidic, carboxylate-enriched hydrophilic motif is hypothesized to mediate strong electrostatic interactions with the HAp substrate. This affinity effectively anchors the peptide at lattice defect sites, transforming them into thermodynamically stable templates for heterogeneous mineral nucleation [17, 39]. Concurrently, the contiguous hydrophobic segment exerts a critical spatiotemporal regulatory role. Rather than inducing rigid molecular aggregation, this domain maintains the peptide’s overall flexibility, functioning as a spatial buffer that regulates the distance between nascent mineral nuclei. This mechanism potentially prevents the chaotic agglomeration of calcium phosphate clusters, thereby guiding ordered, biomimetic crystal growth [40]. Ultimately, the integration of deep infiltration, targeted calcium-anchoring and regulated nucleation spacing offers a cohesive mechanistic explanation for the superior performance of CaAm-P19.

To date, various amelogenin-derived peptides, including LRAP, TRAP, ADP-family peptides and synthetic self-assembling peptides (e.g., P11-4), have been explored for enamel remineralization. However, a ubiquitous limitation is that their reparative efficacy is largely confined to the superficial enamel layer, as these peptides rely on a singular mode of mineral induction. For instance, while LRAP and full-length amelogenin exhibit excellent HAp affinity, their large molecular sizes severely restrict subsurface diffusion [41]. Similarly, TRAP and ADP-family peptides effectively promote surface crystal deposition but lack the capacity to orchestrate homogeneous mineral redistribution within deep lesions [42, 43]. Conversely, synthetic peptides like P11-4 demonstrate reliable self-assembly and nucleation, yet their clinical efficacy remains highly susceptible to ambient pH fluctuations [44]. Against this backdrop, CaAm-P19 presents distinct comparative advantages: it inherits the innate HAp-binding capacity of the amelogenin C-terminus [45–47], while its short-chain architecture guarantees superior diffusion kinetics compared to both full-length amelogenin and the 59-residue LRAP, creating optimal structural prerequisites for deep enamel repair.

The intervention of CaAm-P19 also significantly enhanced the mechanical properties of demineralized enamel, though differing remineralization patterns were observed compared to conventional fluoride treatment. While Micro-CT quantified a superior restoration of volumetric mineral density in the CaAm-P19 group, *SMH* assays revealed no statistical disparity between CaAm-P19 and fluoride. This divergence stems from the fundamental mechanistic differences between the two agents. Fluoride rapidly induced the precipitation of a hyper-mineralized, fluorapatite-enriched surface zone, effectively restoring superficial microhardness. However, this rapid surface pore occlusion restricts subsequent ion diffusion, leaving the deeper lesion regions insufficiently mineralized [48–50]. In contrast, CaAm-P19 infiltrated the entire lesion depth, mediating a homogeneous biomimetic deposition that enhances the global volumetric mineral content. It is worth noting that the 1 mmol/L NaF (≈20 ppm F^−^) utilized in this study is lower than standard clinical concentrations. This conservative dosage acknowledged that excessive fluoride application not only raises the risk of fluorosis but also functioned primarily via demineralization inhibition rather than promoting deep mineral gain [51]. Furthermore, unlike the transient clinical application of fluoride varnishes or dentifrices, fluoride was continuously present in our mineralizing solution. This sustained, low-dose regimen was strategically selected to establish a detectable remineralization baseline while intentionally minimizing rapid surface hyper-mineralization that could obscure the evaluation of deep mineral recovery [43, 52, 53].

While the current study provided valuable insights into CaAm-P19-mediated enamel biomineralization, several limitations must be acknowledged. First, bovine enamel was employed as an alternative substrate. Although widely validated for *in vitro* remineralization studies due to its histological similarities to human enamel [54, 55], inherent differences in baseline mineralization levels, prism packing density and organic matrix content necessitated caution when extrapolating these results to human clinical scenarios [56, 57]. Second, the static *in vitro* models could not fully recapitulate the complex, dynamic intraoral microenvironment, which involves salivary buffering, proteolytic enzymes, dynamic pH cycling and polymicrobial biofilm challenges [58]. Additionally, while PDLSCs were employed to confirm preliminary cytocompatibility, they were not the primary cell types exposed during oral remineralization therapies [30]. Therefore, subsequent biological evaluations should incorporate more physiologically relevant cell lines, such as gingival fibroblasts and oral epithelial cells [59]. Future investigations focusing on peptide stability against salivary proteolysis, *in situ* intraoral models and long-term remineralization efficacy are imperative to validate the translational potential of CaAm-P19.

## Conclusion

In summary, CaAm-P19, a 19-residue amelogenin-derived peptide, demonstrated a stable yet flexible random-coil conformation that facilitated uniform binding to demineralized enamel and profound penetration into subsurface lesions. It successfully orchestrated the ordered deposition of calcium phosphate crystals, significantly restoring both *SMH* and volumetric mineral density while exhibiting excellent cytocompatibility profile. These foundational *in vitro* findings established CaAm-P19 as a highly promising biomimetic candidate for noninvasive enamel therapeutics. Moving forward, transitioning from these static models to dynamic *in vivo* evaluations will be essential to validate its long-term remineralization efficacy, biological safety and clinical translational potential.

## Supplementary Material

rbag123_Supplementary_Data
