# Supplementary material for: CaAm-P19: a novel amelogenin-derived peptide for enamel remineralization
Source: Regen Biomater. 2026 Jun 10;13:rbag123. doi: 10.1093/rb/rbag123 (PMC13310036; doi:10.1093/rb/rbag123)

**Supplementary Table 1.** Secondary structure quantification of CaAm-P19 alone or with Ca^2+^ using BestSel software.

|  | α-helix (%) | β-sheet (%) | β-turn (%) | Random coil (%) |
| --- | --- | --- | --- | --- |
| CaAm-P19 | 8.2±1.3 | 30.4±2.3 | 14.6±2.3 | 46.8±2.6 |
| CaAm-P19 + Ca^2+^ | 7.9±1.6 | 32.1±4.4 | 14.5±0.85 | 45.5±3.4 |

No statistically significant variations existed among all secondary structure components (*p > 0.05*).

**Supplementary Table 2.** Elemental composition (weight percentage, Wt%) and Ca/P molar ratio of enamel samples determined by EDS.

| Element | Control | Demin | Remin | Fluoride | Peptide |
| --- | --- | --- | --- | --- | --- |
| C (Wt%) | 20.71±3.23 | 43.79±3.09 | 25.02±3.29 | 11.58±1.00 | 10.01±1.49 |
| O (Wt%) | 12.44±3.38 | 27.58±3.75 | 26.53±2.08 | 32.17±3.94 | 23.66±2.53 |
| F (Wt%) | 0.24±0.11 | 0.17±0.02 | 0.1±0.01 | 0.49±0.09 | 0.14±0.02 |
| P (Wt%) | 23.93±1.53 | 11.34±2.04 | 18.35±2.24 | 23.49±2.8 | 24.63±2.91 |
| Ca (Wt%) | 42.7±1.44 | 17.11±0.81 | 29.27±2.58 | 38.2±2.3 | 41.34±3.49 |
| Ca/P | 1.79±0.15 | 1.56±0.32 | 1.62±0.26 | 1.64±0.2 | 1.69±0.16 |

EDS results of five enamel groups (Control Group, Demin Group, Remin Group, Fluoride Group, and Peptide Group), including the weight percentages of key elements (C, O, F, Ca, P) and the calculated Ca/P molar ratio. All quantitative EDS data are presented as mean±standard deviation. No statistical difference was observed in Ca/P among all groups (*p > 0.05*).

**HPLC Analysis report of CaAm-P19**.


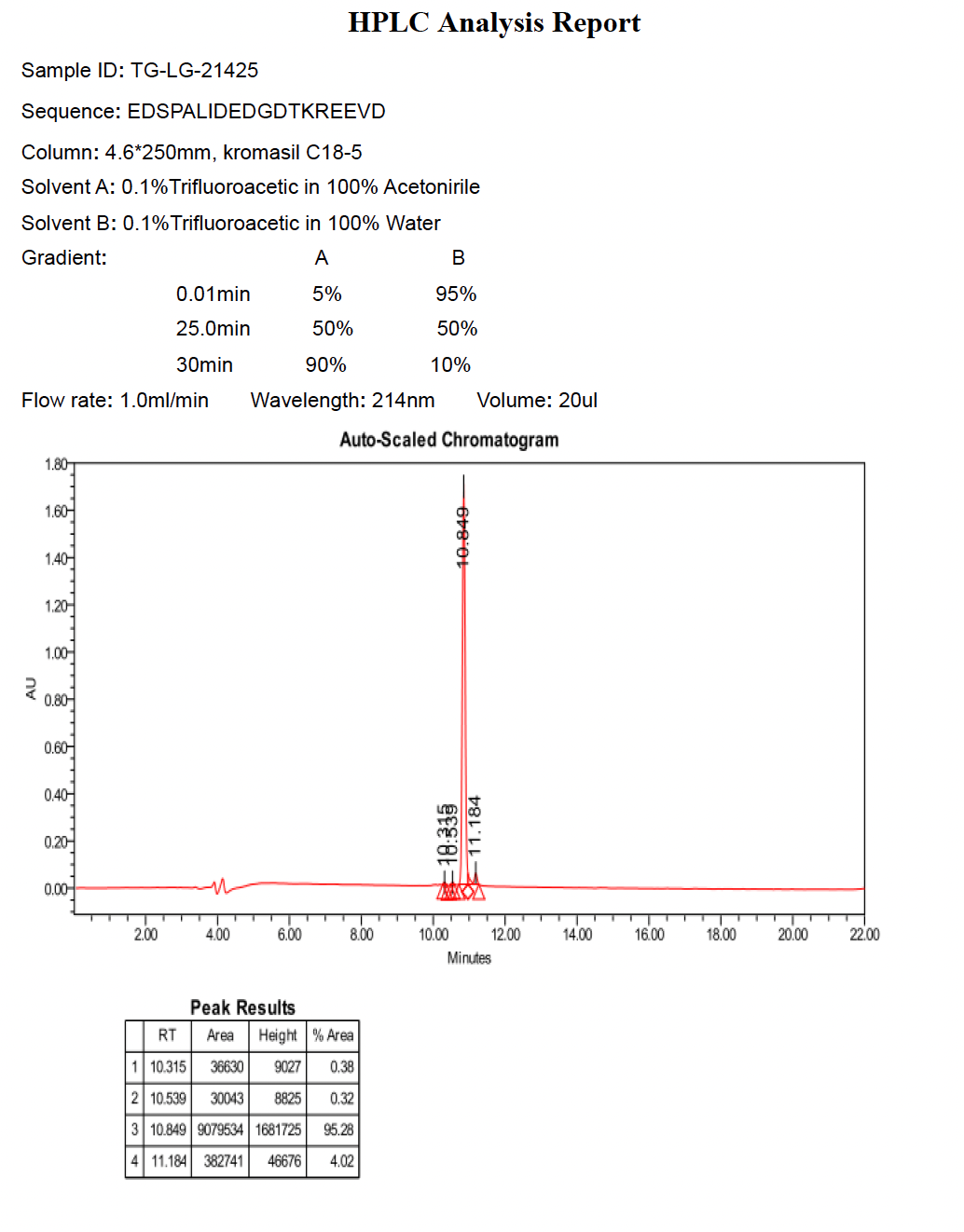


**MS Analysis report of CaAm-P19.**


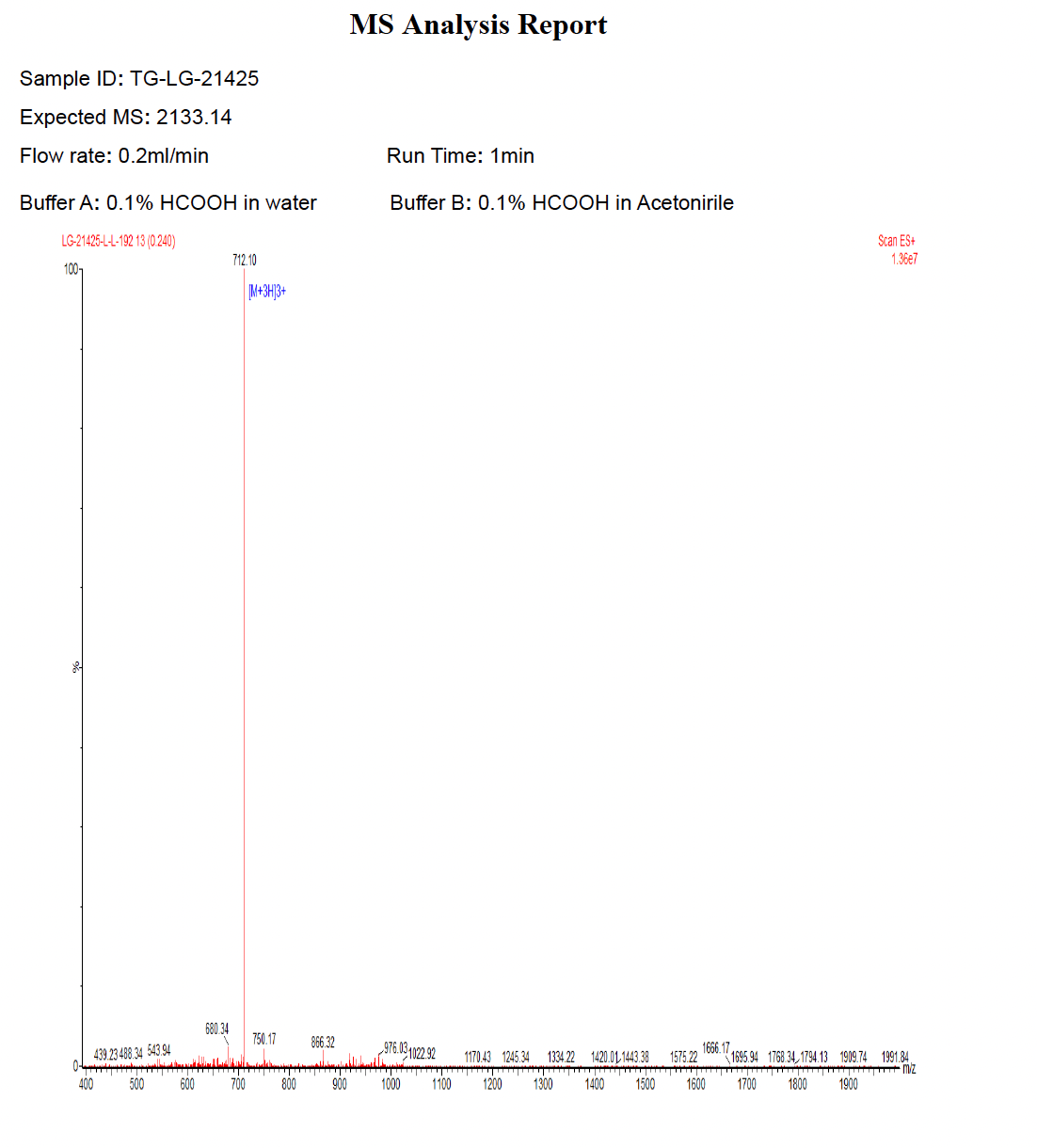


**HPLC Analysis report of FITC-CaAm-P19.**


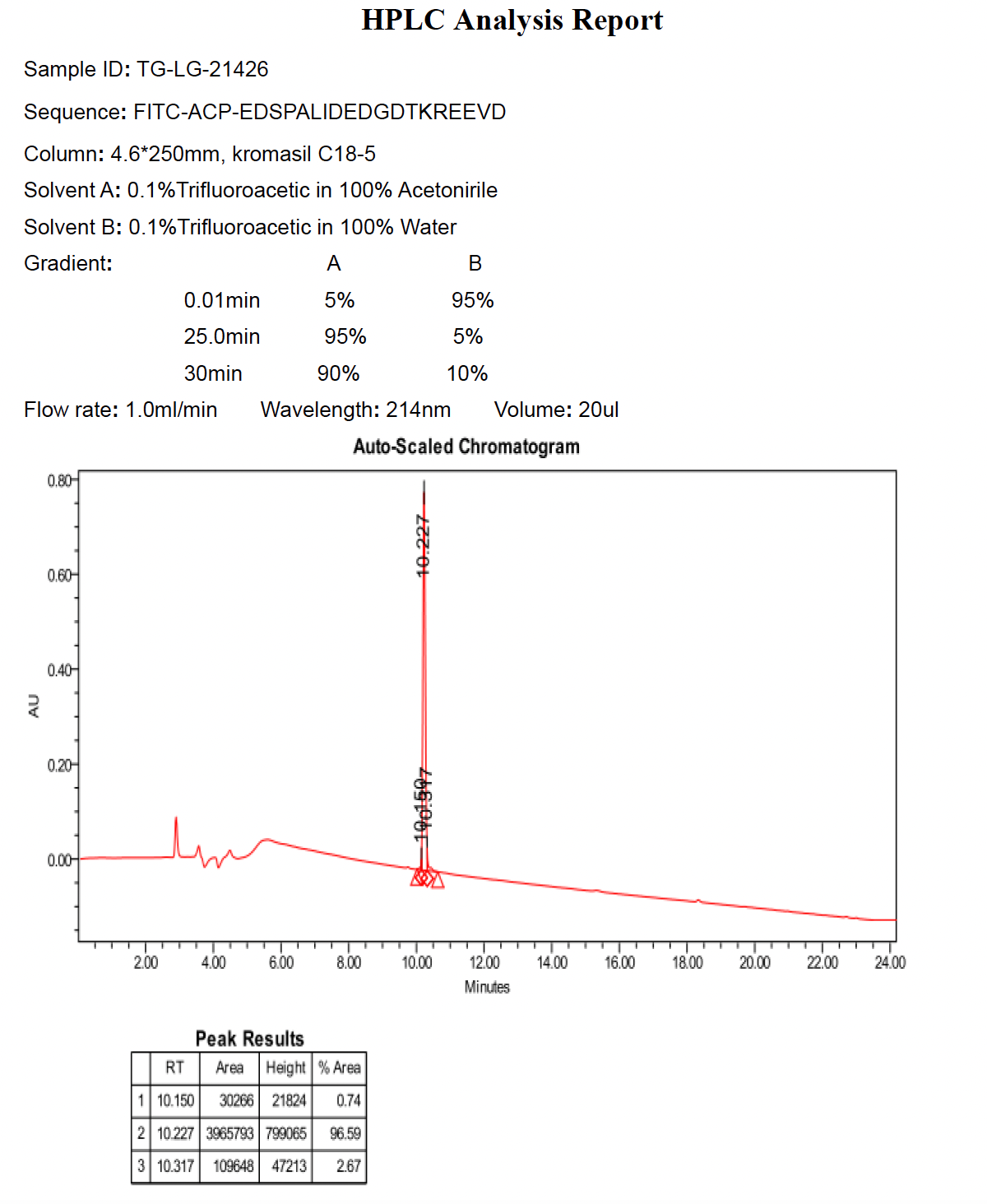


**MS Analysis report of FITC-CaAm-P19.**


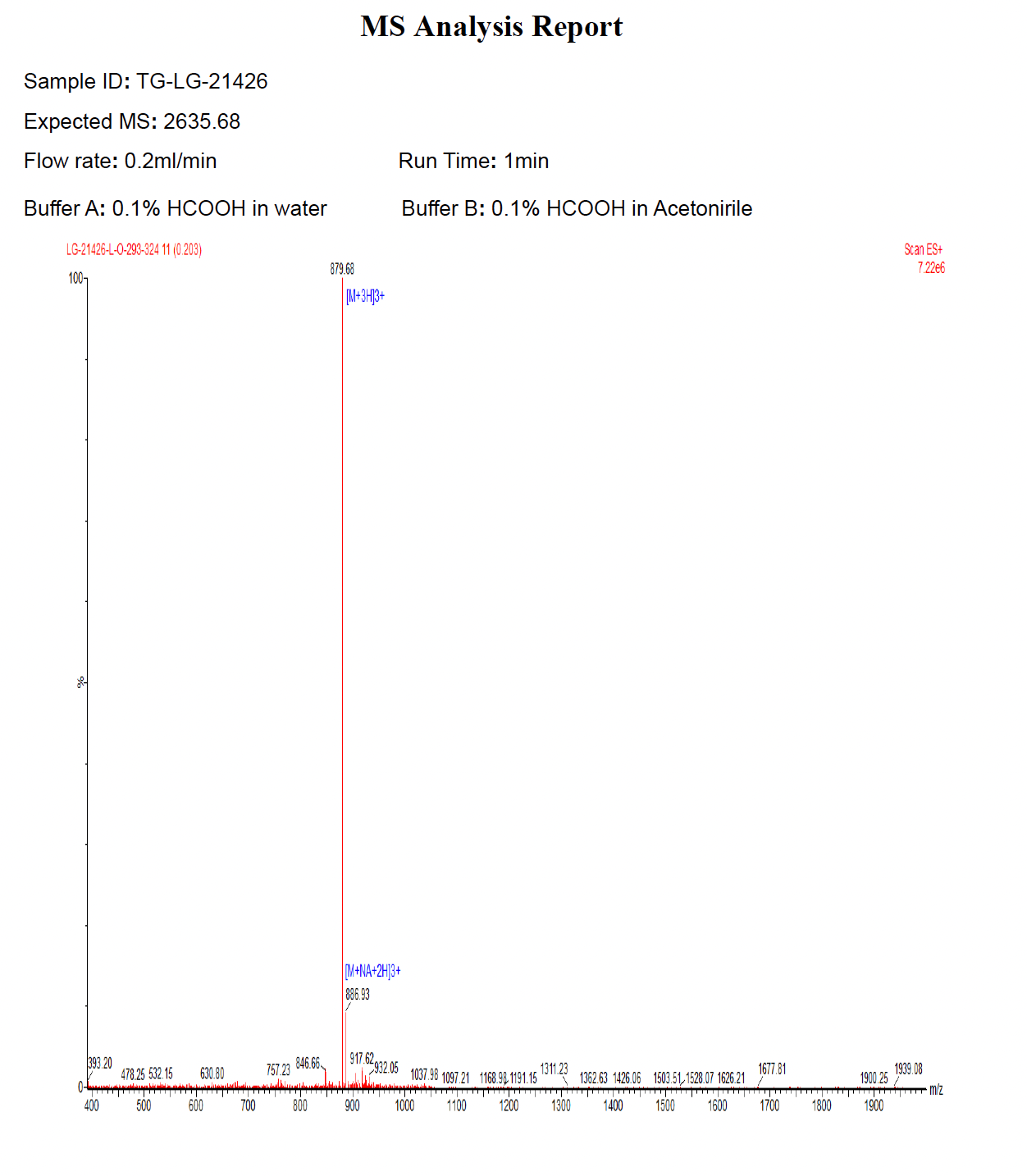

Supplement: rbag123_Supplementary_Data [file rbag123_supplementary_data.docx]
